# Avoiding inconsistencies over time and tracking difficulties in Applied Biosystems AB1700™/Panther™ probe-to-gene annotations

**DOI:** 10.1186/1471-2105-6-307

**Published:** 2005-12-22

**Authors:** Sebastian Noth, Arndt Benecke, systems epigenomics group

**Affiliations:** 1 Institut des Hautes Etudes Scientifiques – Institut de Recherches Interdisciplinaires, CNRS/INSERM, 35, route de Chartres, 91440 Bures sur Yvette, France

## Abstract

**Background:**

Significant inconsistencies between probe-to-gene annotations between different releases of probe set identifiers by commercial microarray platform solutions have been reported. Such inconsistencies lead to misleading or ambiguous interpretation of published gene expression results.

**Results:**

We report here similar inconsistencies in the probe-to-gene annotation of Applied Biosystems AB1700 data, demonstrating that this is not an isolated concern. Moreover, the online information source PANTHER does not provide information required to track such inconsistencies, hence, even correctly annotated datasets, when resubmitted after PANTHER was updated to a new probe-to-gene annotation release, will generate differing results without any feedback on the origin of the change.

**Conclusion:**

The importance of unequivocal annotation of microarray experiments can not be underestimated. Inconsistencies greatly diminish the usefulness of the technology. Novel methods in the analysis of transcriptome profiles often rely on large disparate datasets stemming from multiple sources. The predictive and analytic power of such approaches rapidly diminishes if only least-common subsets can be used for analysis. We present here the information that needs to be provided together with the raw AB1700 data, and the information required together with the biologic interpretation of such data to avoid inconsistencies and tracking difficulties.

## Results

Studying the cellular transcriptome and its dynamics using microarray technology has become a common place application in modern biomedical research [1]. Dedicated databases [*i.e*. [2-4]] store several hundreds of individual microarray datasets and are growing exponentially. Many different commercial and research originating microarray formats and platforms are being used [2-4]. Since microarray technology currently can not be used to determine absolute expression levels of genes, comparative analysis of transcriptome data across different biological conditions is challenging. Cross-platform comparisons can only be carried out if coherent mapping of genes between the platforms and their particular probe-to-gene and gene-to-genome annotations can be achieved. The public microarray databases are very valuable as here standard formatting and annotation procedures are being imposed, thereby rendering the individual microarray experiments useful beyond the immediate purpose they were acquired for [4,5].

Since both the genome sequences as well as individual gene annotations are subject to constant discovery-driven change, probe-to-gene annotations undergo frequent revisions. Such revisions of the initial probe-to-gene mapping lead to inconsistencies if they are not well controlled [6]. Today any dataset requires to be published together with the probe-to-gene annotation used for biological analysis or an explicit reference to a static open-source of such. Unfortunately, this is not state-of-the-art yet, and we join in the call for more sensitivity towards this issue [6].

We show here that for Applied Biosystems AB1700 Genome Survey Arrays the same contention of potential inconsistencies in the probe-to-gene annotations holds true if several precautions are not taken.

**Figure 1:**
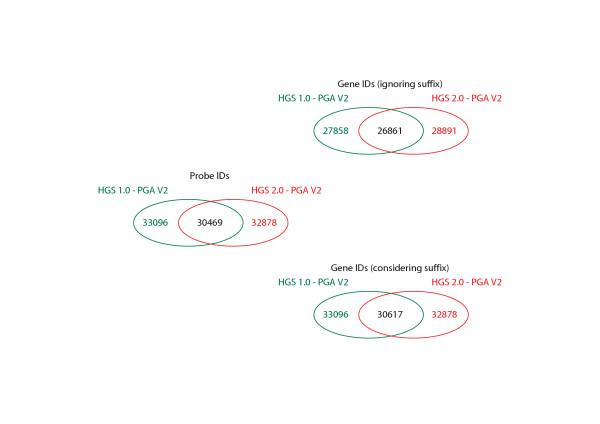
**Venn diagrams comparing the different unique probe and gene sets according to probe-to-gene annotation releases and array versions**. For explanation on gene ID suffixes, please refer to paragraph *5. Obsolete gene IDs and nomenclature suffixes*. A1.0 = Human Genome Survey Array 1.0; A2.0 = Human Genome Survey Array 2.0; V1 = probe-to-gene annotation release 1; V2 = probe-to-gene annotation release 2.

Applied Biosystems AB1700 (ProdNo: 4338036) technology [7], has only recently been commercialized and applied to biomedical research [8]. Design particularities such as the use of 60 mer oligonucleotide probes and the use of chemiluminescence as means of signal detection result in greater sensitivity as comparable commercial platforms. Until present microarray slides for human, mouse, and rat have been developed [7,9-11]. For the Human Genome Survey Arrays (HGS) already a second generation of slides are being commercialized (2.0: ProdNo: 4359029, first release 1.0: ProdNo: 4337467), which contain probes for 29098 individual genes of which >8000 are not covered by other commercial solutions [9] (Figure 1).

The probe-sets as well as the probe-to-gene annotations (PGAs) for AB1700 technology are revised regularly. In order to assure correct and unambiguous interpretation of AB1700 data, we incite particular attention to the following aspects:

### PGA revisions, unless tracked and conserved by the user lead to inconsistencies and ambiguities in the interpretation of AB1700 data

Significant changes between probe-to-gene releases and microarray versions in Applied Biosystems AB1700 data are also observed. The conclusions drawn from the very same experimental dataset will differ depending on the particular PGA version used. In order to retain transparency, the microarray data need thus to be annotated with the PGA version used for interpretation. Currently, AB1700 PGA files are not date-stamped and simply replaced by revised versions in the *AB Gene Expression System Software *[12] once those become available. The AB1700 user has therefore to keep copies of back-versions and a mapping of datasets to PGA files. This problematic has been previously discussed for an analogous case [6].

### AB1700 PGAs are not publicly available, and the PANTHER web-source can not be used for third party verifications

Third parties have no direct access to the AB1700 PGA files. Independent verification of published results could thus only be achieved using the Applied Biosystems PANTHER web-source which is similar in function to NetAffx [13-16]. The user hereby can upload, temporally store, and analyze datasets containing gene or probe IDs and associated signal measurements. PANTHER's use of Hidden Markov Models (HHMs) and protein-family trees clearly provides for significant insights into the nature of the biologic problem studied, and is frequently used by research.

PANTHER internally operates on a continuously updated probe-to-gene annotation table, whereas the PGA releases are communicated discontinuously. Today there is no way of tracking and/or recording the continuous updates. PANTHER also contains only information on protein coding genes. The completeness of the internal PGA is further compromised by excluding GenBank-only mRNAs as they usually do not contain associated protein information. By consequence, published lists of gene or probe IDs with associated PANTHER pathways analyses are ambiguous, potentially incomplete, and the interpretation is not necessarily verifiable by a third party.

### Several probes for a single gene

Probe design for the AB1700 has been guided by the idea to have a single probe targeting all isoforms of a given gene. This is, due to alternative splicing, alternative promoter usage, and likely also to annotation errors, not possible for about 13% of all HGS V2.0 represented genes, which are consequently quantified using up to ten different probes on the array [9-11]. The probe signal intensities are determined individually and need to be kept separate during primary analysis of the data [12]; a challenge for automated data analysis, as the existence of multiple probes for a single gene is not evident from the gene ID or probe ID alone. A look-up table needs to be compiled in order to establish proper mapping between any given gene ID and its single or multiple probes for every new PGA release [see Additional file 1] [see Additional file 2]. Finally, the logic used to integrate the different signals for a single gene needs to be communicated in order to achieve transparency for third parties.

### Several gene IDs for a single probe

Many spotted probes are also not mono-specific for a single gene. In cases of significant cross-reactivity, *e.g*. with closely related members of a gene family, not only a primary gene ID is listed in the PGA tables but in addition, as a separate entry, a list of alternate gene IDs the probe cross-hybridizes with. The existence of multiple transcripts hybridizing to a single probe is obviously not systematic, but also not appreciable from the probe ID nomenclature. Hence a dedicated look-up table needs to be generated.

### Obsolete gene IDs and nomenclature suffixes

In the PGA table a specific column indicates whether the relationship between a given probe ID and the primary gene ID is still valid or has become obsolete during annotation revisions. This information should be applied retroactively to previous annotations of a dataset, and obsolete gene IDs replaced by the current ones if they are available. A number of probes also do no longer match to any known gene. For coherence reasons the primary gene ID listed in the PGA table corresponds to last previously valid entry, and the status is set to obsolete. Curation status of gene annotations is indicated by attaching a version number (suffix) to the gene ID. The probe-to-gene annotation tables provided by the manufacturer list as primary gene ID for 28366, or greater 97% of all represented genes, the Celera Genomics gene nomenclature ID. Comparison with other platforms and publicly annotated genes is hence cumbersome, but since public IDs are also provided for the 22271 transcripts that can be matched, possible. A total of 23300 gene IDs (PGA V2), or >70% the Celera Genomics nomenclature, is suffixed (*i.e*. hCG123456.*4*). A single gene ID can be found within a single release on a single array associated with multiple probes, only differing in the annotation/curation status suffix (a total of 23 gene IDs concerned in annotation release V1; a total of 44 gene IDs concerned in release V2, [see Additional file 1] [see Additional file 2]). The highest running suffix thereby indicates the current state of the art. Lower suffixes correspond to obsolete annotations, and no further information or updates are provided for those gene IDs. The PANTHER database retains for analysis only the highest suffixed gene ID in the submitted set of gene IDs, however without indicating the precise numerical value as they are truncated (Table 1). This practically excludes the option of using PANTHER with sets of gene IDs rather than probe IDs. Moreover, yet another PGA version-specific look-up table needs to be compiled to assure that always the gene ID with the highest ranking suffix is used for the biological interpretation of the data based on gene IDs. The potential confusion is illustrated with the example of PANTHER HMM scores for the Mitogen Activated Protein Kinase Kinase 3 – MAPKK3, which possesses four (V1) or five (V2) associated probes and two gene ID curation versions (Table 2). Given above, since PANTHER is continuously updated, we suggest that submission files are generated and downloaded whenever PANTHER is used with AB1700 data, and that these files are carefully stored together with the biologic or statistic interpretation of the data.

**Table 1:** PANTHER annotation versus disseminated annotation releases 1 & 2. The data from the Supplementary Files 1 & 2 were submitted to the PANTHER website either using the probe IDs or the gene IDs. The differences in identification, display, and absentee calls, between the datasets and the annotation releases are due to the fact that PANTHER is continuously updated, only considers protein coding genes, excludes GenBank-only annotated mRNAs, and retains only the gene ID with the highest suffix.

		**Number of unique entries ~**				
**Annotation Version**	**Probe ID/Gene ID set**	**submitted**	***found *by PANTHER**	***displayed *by PANTHER**	***not found *by PANTHER**	**not accounted for**
V1	Probe ID	51	30	29	21	0
	Gene ID	51	44	22	2	5
						
V2	Probe ID	110	65	59	45	0
	Gene ID	110	98	44	0	12

**Table 2:** Probe/Gene IDs for MAPKK3. The different probe and gene IDs corresponding to the two annotation releases (V& and V2) where submitted to PANTHER with the listed results. This table summarizes for a single gene the differences and potential ambiguities when submitting gene IDs rather than probe IDs, and illustrates the effect of curation status (obsolete *vs*. valid) or annotation release. Note that >70% of all gene IDs in the current HGS V2 PGA table carry suffixes, that ~13% of all represented genes have more than one probe ID associated, and that probes often have secondary hits. All of these require different look-up tables to be generated by the user in order to achieve coherency and transparency in the data analysis process.

		**if Probe IDs (V1) are submitted:**		**if the Gene IDs (V1) are submitted:**	
**Probe ID (V1)**	**Gene ID (V1)**	**PANTHER SCORE**	**PANTHER Gene ID**	**PANTHER SCORE**	**PANTHER Gene ID**
235514	hCG1993739	2 × 10E-23	hCG1993739	2 × 10–23	hCG1993739
127877	hCG1993739.1	6 × 10E-80	hCG28371		
182262	hCG1993739.1	3 × 10E-70	hCG1980405		
234450	hCG1993739.1	6 × 10E-58	hCG1997534		
					
		**if Probe IDs (V2) are submitted:**		**if the Gene IDs (V2) are submitted:**	
**Probe ID (V2)**	**Gene ID (V2)**	**PANTHER SCORE**	**PANTHER Gene ID**	**PANTHER SCORE**	**PANTHER Gene ID**

235514	hCG1993739	2 × 10E-23	hCG1993739	2 × 10–23	hCG1993739
127877	hCG1993739.2	6 × 10E-80	hCG28371		
182262	hCG1993739.2	3 × 10E-70	hCG1980405		
234450	hCG1993739.2	6 × 10E-58	hCG1997534		
171301	hCG1993739.2	-	-		

## Conclusion

The importance of unequivocal annotation of microarray experiments is evident. The analytical power of novel technologies such as the AB1700 platform from Applied Biosystems [7] certainly could be curtailed by incorrect annotation and failure to statically associate the correct annotations with the data.

In order to maintain a maximum of transparency and consistency, we conclude that such microarray data can only be analyzed based on probe IDs rather than gene IDs, and need to be supplemented with the following information for purposes of publication and reanalysis: (i) the microarray version, (ii) the probe-to-gene annotation release used, (iii) how multiple probes for a single gene, and (iv) how probe cross-reactivity have been considered. In case PANTHER was used for analysis: (v) which probe and gene corresponds to the HMM scores, and finally (vi) a submission record-file generated by PANTHER at the same time the statistical and biological analysis was performed.

## Authors' contributions

SN and AB participated in the analysis of AB1700 data and their annotations, the computations, and manuscript preparation. AB has coordinated this study. Both authors have read and approved the final manuscript.

## Supplementary Material

Additional File 1 As discussed in the text, not only multiple probes can exist for a single gene, but also multiple gene IDs. These gene IDs differ by a numerical extension or suffix. They represent the curation status of the gene annotation. Numerically lower suffixes do not have any further annotation information in the probe-to-gene annotation releases, however, are retained for "coherence" reasons. This table lists all obsolete probe ID *vs*. gene ID annotations. Such look-up tables need to be generated by AB1700 users for each PGA [see Additional file 2]. Click here for file

Additional File 2 This file collects the same information as [see Additional file 1] just for the probe-to-gene annotation release 2. Click here for file
